# Sagittal sequence and clinical efficacy of cervical disc replacement and hybrid surgery in the treatment of cervical spondylotic myelopathy: a retrospective study

**DOI:** 10.3389/fsurg.2023.1265349

**Published:** 2024-01-05

**Authors:** Bin Zheng, Shuai Xu, Tianliang Lu, Yonghao Wu, Haoyuan Li, Chen Guo, Liu Haiying

**Affiliations:** ^1^ Spine Surgery Department, Peking University People’s Hospital, Beijing, China; ^2^ Orthopedics Department, The Coal Central Hospital of Shanxi Province, Taiyuan, Shanxi, China

**Keywords:** cervical disc replacement, hybrid surgery, anterior cervical discectomy and fusion, cervical spondylotic myelopathy, sagittal sequence

## Abstract

**Background:**

Hybrid surgery (HS) combines anterior cervical discectomy and fusion (ACDF) with cervical disc replacement (CDR) is gradually being more frequently implemented, but there are few studies reporting the safety and effectiveness of hybrid surgery in three levels cervical spondylotic myelopathy.

**Methods:**

The clinical and radiographic data of patients with three-segment cervical spondylosis, who underwent CDR, ACDF and HS in our hospital from February 2007 to February 2013 were analyzed. The Visual Analog Scale (VAS), Japanese Orthopedic Association (JOA) and Neck Disability Index (NDI) were used to evaluate the clinical efficacy post surgery. Cervical spine x-rays were conducted to assess ROM, CL, T1S and relevant outcomes.

**Results:**

A total of 94 patients were included in the study: 26 in the CDR group, 13 in the HS1 group, 31 in the HS2 group, and 24 in the ACDF group. Most patients in the CDR group were younger. There was no difference in the follow-up duration, blood loss volume or surgery time (*P* > 0.05). Four groups reported improvements in JOA and NDI scores compared to baseline. There was no significant difference in the final JOA, final NDI or recovery rate among the 4 groups. The final ROM was smaller in the ACDF group than in the other 3 groups. There was no difference among the four groups in the final UROM, final LROM or their changes. There was no difference in the final T1S, final SVA or their change among the four groups. All groups showed similar changes in CL and T1S-CL.

**Conclusions:**

There was no difference in the clinical outcomes of ACDF, CDR, or hybrid surgery. CDR can better preserve the mobility of the cervical spine. Neither CDR nor hybrid surgery was significantly advantageous over ACDF in restoring the sagittal sequence in patients with three-level CSM.

## Introduction

The specific surgical procedure for cervical spondylosis may vary depending on the individual case, but the goal is to decompress the spinal cord and stabilize the affected area. Anterior cervical discectomy and fusion (ACDF) has been proven to be effective in relieving symptoms and improving neurological function (1, 2). However, not all cervical spine conditions require fusion. Nonfusion procedures, such as cervical disc replacement, may be considered in specific cases. In CDR, the damaged or diseased disc in the cervical spine is removed and replaced with an artificial disc implant. The goal of CDR is to maintain motion at the treated level while relieving symptoms caused by disc pathology. It aims to preserve natural neck motion at the treated level, which can potentially reduce stress on adjacent discs and decrease the likelihood of adjacent segment disease (3–6). Cervical hybrid surgery is the combination of the principles of ACDF and CDR. Hybrid surgery (HS) allows a tailored approach, where the surgeon can address the specific needs of each cervical level based on the pathology and individual patient considerations (2, 7, 8). Moreover, patients who undergo three-level hybrid surgery may experience biomechanical differences between a single cervical artificial disc combined with two-level interbody fusion devices and a two-level total disc replacement combined with a single-level interbody fusion device.

The sagittal alignment of the cervical spine plays a crucial role in maintaining normal biomechanics and preserving cervical mobility. Previous studies have suggested that both ACDF and hybrid surgery can restore local cervical lordosis and influence overall cervical alignment (9–12). However, this conclusion has not yet been established for three-level surgeries. The aim of this retrospective study is to compare multiple indicators, including the postoperative relief of clinical symptoms, overall mobility of the cervical spine, and sagittal position parameters, in patients diagnosed with CSM who underwent a three-level procedure with CDR, two types of HS, and ACDF and were followed up for a mean duration of 11 years.

## Methods

### Patient enrollment

The series consisted of patients diagnosed with CSM who underwent (1) cervical disc replacement, (2) hybrid surgery involving either 2-level CDR (HS1) or 1-level CDR (HS2), and (3) ACDF. The study was conducted between February 2007 and February 2013 at a single center in Peking University People's Hospital. Informed written consent was obtained from all patients before their enrollment in the study.

The study's inclusion criteria were as follows: (I) patients who had undergone more than 6 months of conservative treatment and needed surgery and (II) patients who had consecutively undergone three-level CDR, HS1, HS2 or ACDF. (III) Patients with complete radiographic and clinical outcome data. The exclusion criteria were as follows: (I) patients with unclear radiological parameters for measurement, (II) patients with a history of previous cervical surgery, and (III) patients who had undergone cervical spine surgery for trauma, malignancy, or infection. (IV) patients who were lost to follow-up or who died.

All patients underwent CDR, HS1, HS2 or ACDF using the standard Smith-Robinson approach by the same senior surgeon. The Prodisc-C (Depuy Synthes) artificial disc was used, and the polyether-etherketone cage used was the MC + (LDR Medical, France).

### Clinical and radiological outcomes

Radiological parameters were assessed using lateral x-ray imaging in both flexion-extension and neutral positions. These assessments were conducted by two spine surgeons who were independent of the surgical team and did not participate in surgeries. (I) T1 slope angle (T1S): The T1 angle is defined as the angle formed by two lines: the first line extends from the center of the T1 endplate perpendicular to the T1 endplate, while the second line extends from the center of the T1 endplate to the upper aspect of the sternum. (II) Cervical lordosis (CL): Cobb angle method; a line is drawn from the inferior endplate of C2 to the inferior endplate of C7. Perpendicular lines are then created between these two lines, forming an angle that represents cervical lordosis (CL). (III) Sagittal vertical axis (SVA): In the measurement of the C2-C7 sagittal vertical axis on an upright cervical spine lateral radiograph, a plumb line is dropped from the centroid of C2, and the posterior-superior aspect of C7 is used as a reference point. (IV) Range of motion (ROM): Cervical dynamic radiographs are used to assess the mobility of the cervical spine.

Clinical outcomes: The clinical outcomes in this study were assessed using the Neck Disability Index (NDI) and the Japanese Orthopedic Association (JOA) score. These measures were evaluated at three time points: pre operation, post operation, and final visit. The recovery rate of the JOA score was calculated using the Hirabayashi method: Recovery rate (%) = ((postoperative JOA-preoperative JOA)/(17-preoperative JOA)) * 100.

### Statistical analysis

Statistical analysis was performed using SPSS 25.0 software. For continuous data, normality and homogeneity of variance were assessed. Normally distributed data were expressed as the mean ± standard deviation (mean ± SD), while skewed distribution data were represented by the median. Single-factor analysis of variance (ANOVA) was conducted, followed by pairwise comparisons using the LSD-T test for intergroup comparisons. For categorical data, counts (percentages) were used, and the chi-square test or Fisher's exact test in contingency tables was applied. A significance level of *P* < 0.05 indicated statistically significant differences.

## Results

### Demographic characteristics and baseline conditions

**Figure 1:**
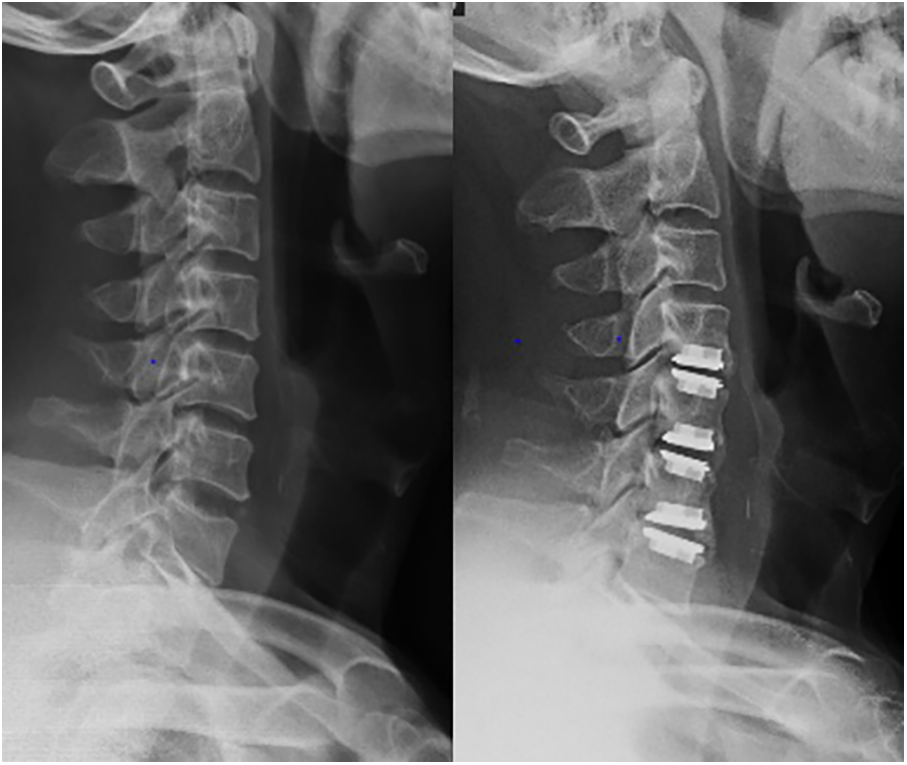
Imaging data of patient with C4–C7 cervical spondylotic myelopathy before and after 3-levels cervical disc replacement treatment.

**Figure 2:**
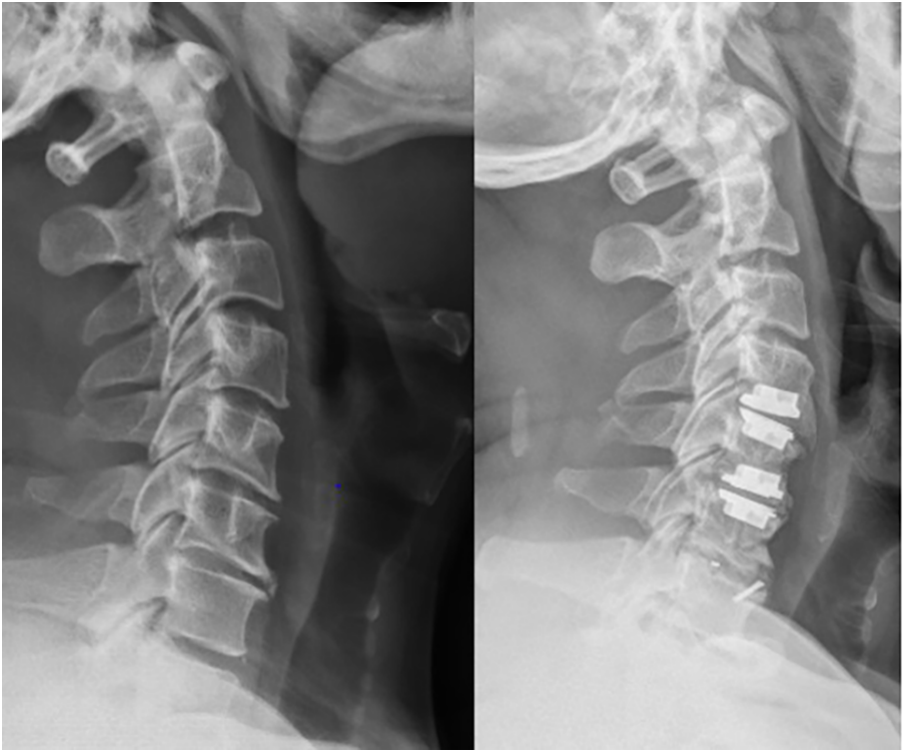
Imaging data of patient with C4–C7 cervical spondylotic myelopathy before and after hybrid surgery treatment.

**Figure 3:**
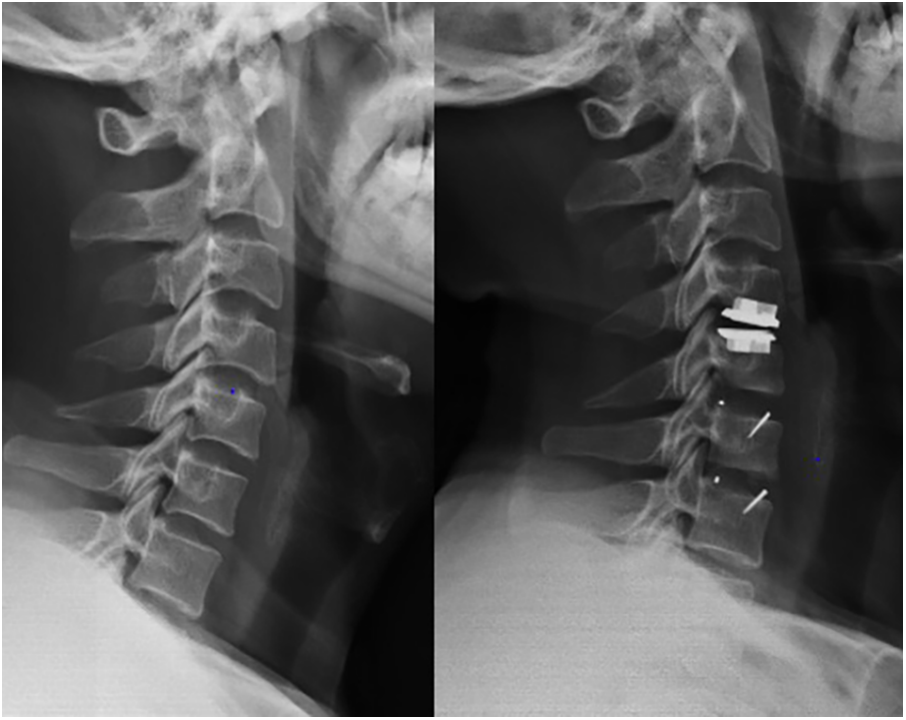
Imaging data of patient with C4–C7 cervical spondylotic myelopathy before and after hybrid surgery treatment.

A total of 94 participants were included in the final analysis. The mean follow-up time was 136.9 ± 7.5 months. Patients in the CDR group were the youngest, while those in the ACDF group were the oldest (*P* < 0.01). There was no difference between the HS1 and HS2 groups. Additionally, there were no significant differences in terms of surgical segment, blood loss volume, or BMI among the four groups. The baseline characteristics are shown in Table 1. Typical comparison imaging before and after the surgery are presented, shown in Figures 1–3.

**Table 1:** Baseline of 4 groups.

	CDR	HS1	HS2	ACDF	*P*
Sample (M/F)	16/10	8/5	14/17	14/10	0.465
Age	43.3 ± 9.3	56.0 ± 11.3	56.3 ± 7.5	64.6 ± 8.6	<0.01
BMI	25.6 ± 4.4	26.3 ± 1.4	25.0 ± 3.1	24.2 ± 3.5	0.309
Follow-up (month)	136.5 ± 9.3	134.8 ± 5.4	138.6 ± 7.6	136.9 ± 7.5	0.4
Surgery segments					0.94
C3–C6	9	5	11	7	
C5–C7	17	8	20	17	
Blood loss	95.4 ± 56.8	74.6 ± 25.4	94.42 ± 70.8	69.6 ± 41.3	0.263
Surgery time (min)	111.5 ± 29.2	123.1 ± 12.5	108.7 ± 17.3	110.4 ± 22.3	0.099

CDR, cervical disc replacement; HS1, one artificial disc and two cages; HS2, two artificial discs and 1 cage; ACDF, anterior cervical discectomy and fusion.

### Clinical outcomes before and after surgery

The comparative analysis of NDI and JOA score at the baseline and the final follow-up revealed an absence of substantial disparity (*P* > 0.05). The average rate of recovery across the four cohorts under study was 77.8% ± 27.3%. Moreover, a noteworthy statistical distinction in the recovery rates among the four cohorts was not discernible. Nevertheless, all cohorts exhibited a significant enhancement at the final follow-up relative to the baseline, denoting statistical significance (*P* < 0.05).

### Radiographic outcomes in sagittal parameters before and after surgery

There was no difference among the four groups in terms of ROM, UROM, or LROM at baseline or in the changes before and after surgery. During the final follow-up, there was no difference among the four groups in the UROM or LROM. The ACDF group had a smaller final ROM than the other three groups (*P* < 0.05), and there was no difference among the remaining three groups in terms of the final ROM.

At the initiation of the study, no substantial disparities were observed among the four cohorts regarding all radiographic outcomes, except for CL and T1S-CL. Additionally, there were no significant differences in SVA, T1S and CL or their changes among the four groups at the final follow-up.

CL in the final follow-up in the ACDF group was smaller than that in the other three groups, and there was no difference among the HS1, HS2 and CDR groups (*P* > 0.05). There was no difference in the change in CL among the four groups. This is mirrored in the results pertaining to OCL and T1S-CL. The details are shown in Table 2, in which the sagittal parameters before surgery and at the final follow-up are presented for all four groups. The details are shown in Table 3.

**Table 2:** Clinical outcomes in four surgery groups.

	CDR	HS1	HS2	ACDF	*P*
JOA baseline	12.3 ± 2.7	12.7 ± 1.0	12.3 ± 1.5	12.2 ± 1.4	0.862
JOA final	15.8 ± 1.5	15.7 ± 0.9	16.2 ± 1.0	15.3 ± 1.9	0.150
NDI baseline	37.5 ± 2.7	37.84 ± 2.0	39.5 ± 3.8	37.9 ± 2.5	0.070
NDI final	14.8 ± 4.9	12.5 ± 2.0	12.7 ± 3.3	13.6 ± 8.4	0.417
RR (%)	77.9 ± 31.8	69.9 ± 19.1	86.8 ± 18.1	70.4 ± 33.1	0.098

CDR, cervical disc replacement; HS1, one artificial disc and two cages; HS2, two artificial discs and 1 cage; ACDF, anterior cervical discectomy and fusion; JOA, Japanese Orthopedic Association score; NDI, neck disability index; RR (%), recovery rate (%).

**Table 3:** Radiological outcomes comparison among 4 groups.

	CDR	HS1	HS2	ACDF	*P*
ROM baseline	41.3 ± 17.0	38.5 ± 10.2	42,7 ± 13.1	38.2 ± 13.0	0.573
ROM final	29.1 ± 11.6	29.8 ± 9.0	29.0 ± 8.4	22.0 ± 7.9	0.019
△ROM	−12.2 ± 15.8	−8.6 ± 6.6	−13.7 ± 10.7	−16.2 ± 5.8	0.615
UROM baseline	7.8 ± 5.2	8.6 ± 2.9	9.3 ± 4.4	13.7 ± 17.4	0.157
UROM final	7.4 ± 6.2	7.7 ± 4.5	8.5 ± 7.4	8.5 ± 7.4	0.878
△UROM	−0.3 ± 3.5	−1.0 ± 3.2	−2.0 ± 4.5	−1.9 ± 4.5	0.504
LROM baseline	5.4 ± 8.5	5.1 ± 2.4	7.2 ± 4.8	8.4 ± 7.4	0.325
LROM final	4.1 ± 2.4	5.2 ± 2.3	4.5 ± 3.5	6.9 ± 6.6	0.109
△LROM	−1.3 ± 8.3	0.3 ± 2.2	−2.3 ± 3.8	−1.6 ± 5.6	0.577
SVA baseline	2.0 ± 1.1	1.9 ± 0.6	1.5 ± 1.1	1.8 ± 1.1	0.212
SVA final	1.8 ± 0.9	2.0 ± 1.3	1.7 ± 1.2	2.0 ± 1.2	0.740
△SVA	−0.2 ± 1.3	0.1 ± 1.2	0.1 ± 1.0	−0.1 ± 1.5	0.766
T1S baseline	27.0 ± 7.0	22.8 ± 3.4	23.5 ± 7.8	24.9 ± 9.1	0.257
T1S final	26.4 ± 6.9	27.0 ± 2.1	26.1 ± 7.5	25.6 ± 7.8	0.952
△T1S	0.7 ± 8.7	4.1 ± 2.1	2.7 ± 5.5	0.7 ± 9.7	0.193
CL baseline	18.0 ± 15.5	10.5 ± 15.3	11.1 ± 11.2	8.5 ± 14.7	0.097
CL final	23.9 ± 12.4	19.9 ± 11.4	15.7 ± 7.2	12.7 ± 9.6	0.001
△CL	6.6 ± 11.4	9.3 ± 12.1	4.6 ± 9.9	4.2 ± 10.1	0.491
UCL baseline	5.3 ± 3.1	6.5 ± 5.9	3.2 ± 4.5	4.1 ± 6.6	0.194
UCL final	2.1 ± 1.7	7.3 ± 5.9	3.2 ± 4.5	1.9 ± 4.3	0.001
△UCL	−2.9 ± 2.4	0.8 ± 6.5	−0.1 ± 4.5	−2.2 ± 6.5	0.059
LCL baseline	5.3 ± 2.5	6.9 ± 2.7	5.6 ± 6.1	3.6 ± 7.1	0.310
LCL final	2.4 ± 2.3	5.2 ± 2.4	5.2 ± 5.4	2.8 ± 8.1	0.146
△LCL	−2.9 ± 2.5	−1.7 ± 2.0	−0.5 ± 4.3	−0.8 ± 6.9	0.206
OCL baseline	2.7 ± 3.7	3.9 ± 12.3	7.8 ± 9.0	4.7 ± 10.3	0.177
OCL final	8.1 ± 6.2	12.9 ± 12.2	14.1 ± 7.1	9.7 ± 7.5	0.027
△OCL	5.4 ± 8.2	7.7 ± 6.7	5.9 ± 9.0	5.9 ± 9.3	0.28
T1S-CL baseline	9.1 ± 11.3	12.3 ± 13.7	12.4 ± 10.1	16.4 ± 10.1	0.140
T1S-CL final	1.9 ± 9.0	7.0 ± 9.7	10.4 ± 8.1	12.9 ± 9.9	<0.001
△T1S-CL	−7.2 ± 8.2	−5.3 ± 13.1	−2.0 ± 9.6	−4.3 ± 9.7	0.217

CDR, cervical disc replacement; HS1, one artificial disc and two cages; HS2, two artificial discs and 1 cage; ACDF, anterior cervical discectomy and fusion; ROM, C2–C7 range of motion; UROM, range of motion of upper adjacent segment; LROM, range of motion of lower adjacent segment; SVA, C2–C7 sagittal vertical axis; T1S, T1 slope; CL, cervical lordosis; T1S-CL, T1S minus CL; △, final outcome minus baseline outcome.

## Discussion

Cervical spondylosis myelopathy encompasses the symptoms and signs that emerge because of damage to the spinal cord, nerves, and blood vessels, attributable to the degenerative alterations in the cervical intervertebral discs and the ensuing degenerative changes in the facet joints (13–15). Through anterior cervical surgery, it is feasible to excise the intervertebral discs and proliferative bone spurs from the anterior aspect, thus mitigating the compression on critical structures, such as the spinal cord, nerve roots, and vertebral arteries. Moreover, the anterior surgical approach facilitates the restoration of the intervertebral space's height, the enlargement of the intervertebral foramen to alleviate compression on the nerve roots, and the reinstatement of the cervical spine's normal curvature (16–18). The cervical spine's sagittal balance represents an outward indication of its biomechanical stability; any sagittal imbalance signifies that there have been shifts in the biomechanics of the cervical spine. The biomechanical attributes of the cervical spine are governed through muscular modulation, playing a critical role in the transmission of cranial loads and the protection of the spinal cord. Consequently, there is escalating attention toward the restoration of the cervical spine's sagittal parameters and biomechanical balance. The underlying aim of artificial cervical disc replacement is to sustain the mobility of the surgical segment while concurrently preserving the cervical spine's stability, with the objective of achieving biomechanical equilibrium and reducing the incidence of adjacent segment disease.

With similar demographic profiles (excluding age), clinical baselines, and radiological indicators, the choice of surgical approach may be associated with age and economic status. Younger patients tend to opt for cervical disc replacement to preserve cervical mobility (19), and the higher cost of cervical disc replacement is more favored by higher-income individuals (20, 21). In our study, a similar trend was observed, with a higher proportion of younger patients opting for cervical disc replacement, consistent with previous research findings. However, due to privacy considerations, we did not stratify patients based on their income. Moreover, in retrospective studies, bias is unavoidable. Our study not only documented the final outcomes for patients but also incorporated changes in indicators over time to mitigate further bias.

In this study, the clinical effectiveness of CDR, HS1, HS2 and traditional ACDF surgery in treating multilevel degenerative cervical spondylosis was assessed through a statistical analysis of NDI and JOA scores for the patients involved. The clinical metrics indicate that the patients’ conditions were improved following all four surgical procedures, suggesting that each of the four surgeries is efficacious in ameliorating the symptoms associated with cervical spondylotic myelopathy. This analysis revealed no significant differences in JOA and NDI scores from before the surgery to the final follow-up. Furthermore, no differences were observed in the rates of symptom relief among the four groups at the final follow-up. The results of the analysis suggest that the clinical outcomes for treating multilevel degenerative CSM are similar across the four surgical approaches. There was also no difference in the recovery of neurological symptoms. This is because in both cervical disc replacement and anterior cervical decompression and fusion, the initial phase of the surgery is focused on comprehensive decompression of the compromised area, aiming to maximize the recovery of nerve and spinal cord functionality. There was no difference among the four surgical techniques in terms of their ability to decompress the spinal canal and nerves. They all provide reliable long-term decompression effects.

This study investigates the effects of four distinct surgical techniques on the physiological ROM in the cervical spine. The ROM is assessed in terms of flexion and extension along the sagittal plane, accounting for both the cervical spine as a whole and its adjacent segments, namely, superior and inferior. The key findings from the analysis revealed that prior to surgery, there was no significant variation in ROM across the cervical spine and its adjacent segments among the four groups under consideration. However, after the surgeries, there was a noted reduction in the ROM in the cervical spine and the neighboring segments in all four groups when compared to their preoperative status. Moreover, there was no discernible difference in the overall cervical spine ROM between the CDR group and HS group. Radiological evaluations confirmed that despite a marked decrease in ROM in the flexion and extension of the cervical spine in both the HS and CDR groups post surgery, they maintained higher ROM than the ACDF group. This suggests that the use of artificial intervertebral discs contributes to preserving the ROM. Furthermore, the UROM and LROM were similar across all four groups. However, the ACDF group exhibited a reduced overall cervical spine ROM compared to the other groups. This indicates that the artificial disc predominantly mitigates the impact of the surgery on cervical spine mobility by preserving the ROM of the surgically treated segment. The material employed in cervical disc replacement is evolving to closely emulate the natural intervertebral disc, which is fundamental for spinal motion and shock absorption. In contrast, the anterior fusion procedure involves the fusion of vertebrae and impacts the ROM in the cervical spine, particularly in the surgically treated area. Hybrid cervical surgery, comprising both fusion and disc replacement, might restrict ROM in certain regions but uphold or enhance ROM in others. Its cumulative impact on the cervical spine ROM is more favorable than that of fusion alone. However, its ROM is comparable to that of replacement alone, which could be attributed to the artificial disc's superior capacity to preserve ROM compared to the restrictions imposed by bony fusion. Grasso and Lu's study also reported advantages in preserving overall mobility with hybrid surgery and disc replacement (7, 22). Previous research has suggested that CDR and HS have an edge in conserving motion at adjacent segments (23), although some studies have reported no significant differences (24, 25). Our research indicates a similar absence of disparities, in line with Matsumoto's perspective (26). The surgery primarily addresses the responsible cervical segment, preserving the adjacent segments and causing no iatrogenic effects on neighboring vertebrae. Besides, the ultimate mobility of patients does not significantly impact their daily life. Even within the ACDF group, it may not be necessary to increase motion at adjacent segments to enhance the overall segmental mobility, as it maintains good stability. Nevertheless, further investigation with a larger sample size is required in this context.

An excessively large value of SVA may lead to excessive forward tilt of the head, causing the neck muscles to bear a greater burden in order to maintain the balance of the head. This can potentially cause symptoms such as neck pain, headaches, muscle fatigue, and limited activity. After cervical surgery, improvements in the cervical SVA are usually associated with good clinical outcomes, such as relief of patient pain and improvements in quality of life (27, 28). There was no difference in the baseline SVA, final follow-up results, or change values among the four groups, indicating that there was no difference in the ability to restore the SVA among the four surgical groups.

The C2–7 Cobb angle is commonly used to evaluate the degree of lordosis of the cervical spine. In this study, the CL of the cervical spine in the ACDF group was smaller than that in the other three groups at baseline and the final follow-up. This may be related to the relatively older age of patients who chose ACDF surgery. However, there was no difference among the four groups in terms of the correction angle of CL. There was also no difference in the change in OCL among the four groups. Therefore, it can be proven that there is no difference among the four groups in terms of the ability to restore cervical lordosis, and there is no difference in the ability to correct sagittal balance. The C2–7 Cobb angle in patients with myelopathy due to cervical spondylosis is smaller than that in healthy individuals. The normal cervical spine has an anteriorly convex curvature. If it exceeds the normal physiological curvature and becomes posteriorly convex, the cervical intervertebral discs will be subjected to greater pressure (29). This can eventually lead to an increase in pressure within the spinal canal, causing pathological changes in spinal cord nerve cells. Therefore, some researchers believe that maintaining an anterior convex shape in the sagittal plane of the cervical spine is the optimal state. Whether it is a straight or posteriorly convex shape, both can increase the risk of degeneration of the cervical intervertebral discs (30, 31). The results of this study indicate that there is no difference in the ability to restore the lordotic state of the cervical spine among the four surgical methods. Prior research suggests that, compared to preoperative conditions, CDR can maintain the sagittal curvature of the cervical spine, but it can only sustain it rather than reconstruct cervical alignment (32, 33). In this study, significant improvements were observed in all four groups. CL and OCL exhibited significant differences compared to the baseline. This improvement can be attributed to the complete removal of osteophytes, restoration of endplate beds, and the appropriate selection of implants.

The first thoracic vertebra is located at the junction between the cervical spine and the thoracic spine and serves as the base for the skull and cervical spine, reflecting the relationship between the cervical spine and the balance of the entire spinal column. T1S determines the position of the head's center of gravity as well as the amount of its anterior curvature. Researchers have found that a smaller T1S can increase the risk of developing myelopathy due to cervical spondylosis. T1S values are lower than those in healthy individuals (34, 35). The current study reveals that in all four patient groups, the postoperative T1S increased compared to baseline levels, with this change being statistically significant. However, there was no statistically significant difference among the four groups, demonstrating that CDR, HS, and ACDF surgeries have no differential impact on long-term T1S in patients.

T1S-CL is a parameter in the cervical spine deformity classification system proposed by Ames and colleagues in 2015 (36). This parameter combines radiological features of both the cervical and thoracic spine, similar to the lumbar lordosis parameter in cases of thoracolumbar deformities. Currently, there are numerous studies that have confirmed the correlation between T1S-CL and the severity of symptoms in patients with degenerative cervical spine diseases, as well as the efficacy of postoperative outcomes (37–39). When T1S decreases, the cervical spine will compensate by reducing CL, leading to the progression of the cervical curvature toward a straight state. This mechanism change may increase intervertebral stress, thereby accelerating the degeneration of cervical intervertebral discs. Those with a larger T1S need a larger cervical lordosis and more load on the posterior neck muscles and ligaments to maintain horizontal gaze. However, when the posterior neck muscle-ligament complex is damaged, the cervical spine has difficulty bearing the weight of the head, which can easily lead to sagittal imbalance and gradually lead to the appearance of structural cervical kyphosis deformity. Previous research has shown that T1S-CL is closely related to CL, and this relationship is similar to that between pelvic incidence (PI) and lumbar lordosis (LL). A large T1S requires a large LL, and thoracic kyphosis accordingly increases. Similarly, a large PI requires a large LL, and thoracic kyphosis also increases, leading to an increase in T1S and CL. If the overall change in CL with the variation in LL is not sufficient to maintain the position of the head above the pelvis, this will lead to an increase in muscle tension and pain. However, at this point, maintaining horizontal gaze should be sufficient. Our study reveals that there is a difference in the baseline T1S-CL values among the four groups, with the ACDF group showing the highest T1S-CL value, while no difference is found among the remaining three groups. In the final outcome measures, the T1S-CL of all four groups also showed differences, but their change values were comparable. This indicates that there is no difference in the ability of the four surgical methods to restore sagittal balance.

There are some limitations. (I) This was a retrospective study with a small sample of all groups so high-level grade randomized controlled trials or large sample cohort studies are needed. (II) The study only included patients with cervical spondylosis of the spine; therefore, the conclusion may not be applicable to other cervical diseases, such as cervical spondylosis of the nerve root or ossification of the posterior longitudinal ligament. Economic considerations and patient preferences: (III) The studies may not account for the economic considerations and individual preferences of the patients, which could have a bearing on the choice of surgical intervention and the outcomes thereof. The incorporation of economic analyses and patient preference assessments is vital to ensure that the conclusions drawn are reflective of real-world decision-making processes. Lack of Multicentricity: While multicenter studies have the potential to offer a more diversified dataset and a broader patient population, this investigation was limited to a single institution. The generalizability of the findings may thus be constrained. Future studies should consider multicentric collaborations to encompass a wider spectrum of patient demographics and clinical practices.

## Conclusion

In conclusion, CDR, HS1, HS2, and ACDF all demonstrated significant and long-term efficacy in the treatment of three-level CSM. The clinical outcomes among the four groups were comparable. The ACDF group exhibited a reduced ROM compared to the other three groups, while no significant difference in UROM/LROM was observed between the CDR, HS1, and HS2 groups. Neither CDR nor hybrid surgery displayed any significant advantages over ACDF in restoring the sagittal sequence in three-level CSM.

## Data Availability

The original contributions presented in the study are included in the article/Supplementary Material, further inquiries can be directed to the corresponding author.

## References

[1] Chen Y Wang X Lu X Yang L Yang H Yuan W Comparison of titanium and polyetheretherketone (PEEK) cages in the surgical treatment of multilevel cervical spondylotic myelopathy: a prospective, randomized, control study with over 7-year follow-up. Eur Spine J. (2013) 22(7):1539–46. 10.1007/s00586-013-2772-y 23568254 PMC3698331

[2] Li W Zhan B Jiang X Zhou G Li J Wang Y. A randomized controlled study of two different fixations in anterior cervical discectomy of multilevel cervical spondylotic myelopathy. J Orthop Surg (Hong Kong). (2022) 30(3):10225536221118601. 10.1177/10225536221118601 36069629

[3] Lavelle WF Riew KD Levi AD Florman JE. Ten-year outcomes of cervical disc replacement with the BRYAN cervical disc: results from a prospective, randomized, controlled clinical trial. Spine (Phila Pa 1976). (2019) 44(9):601–8. 10.1097/BRS.0000000000002907 30325888

[4] MacDowall A Canto Moreira N Marques C Skeppholm M Lindhagen L Robinson Y Artificial disc replacement versus fusion in patients with cervical degenerative disc disease and radiculopathy: a randomized controlled trial with 5-year outcomes. J Neurosurg Spine. (2019) 30(3):323–31. 10.3171/2018.9.SPINE18659 30641852

[5] Pandey PK Pawar I Gupta J Verma RR. Comparison of outcomes of single-level anterior cervical discectomy with fusion and single-level artificial cervical disc replacement for single-level cervical degenerative disc disease. Spine (Phila Pa 1976). (2017) 42(1):E41–e9. 10.1097/BRS.0000000000001696 28002363

[6] Phillips FM Allen TR Regan JJ Albert TJ Cappuccino A Devine JG Cervical disc replacement in patients with and without previous adjacent level fusion surgery: a prospective study. Spine (Phila Pa 1976). (2009) 34(6):556–65. 10.1097/BRS.0b013e31819b061c 19240664

[7] Grasso G. Clinical and radiological features of hybrid surgery in multilevel cervical degenerative disc disease. Eur Spine J. (2015) 24(Suppl 7):842–8. 10.1007/s00586-015-4281-7 26463866

[8] Hey HW Hong CC Long AS Hee HT. Is hybrid surgery of the cervical spine a good balance between fusion and arthroplasty? Pilot results from a single surgeon series. Eur Spine J. (2013) 22(1):116–22. 10.1007/s00586-012-2486-6 22922801 PMC3540319

[9] Shin DA Yi S Yoon DH Kim KN Shin HC. Artificial disc replacement combined with fusion versus two-level fusion in cervical two-level disc disease. Spine (Phila Pa 1976). (2009) 34(11):1153–9. discussion 60-1. 10.1097/BRS.0b013e31819c9d39 19444062

[10] Zigler JE Sachs BL Rashbaum RF Ohnmeiss DD. Two- to 3-year follow-up of ProDisc-L: results from a prospective randomized trial of arthroplasty versus fusion. Sas J. (2007) 1(2):63–7. 10.1016/S1935-9810(07)70048-8 25802580 PMC4365572

[11] Liu JM Peng HW Liu ZL Long XH Yu YQ Huang SH. Hybrid decompression technique versus anterior cervical corpectomy and fusion for treating multilevel cervical spondylotic myelopathy: which one is better? World Neurosurg. (2015) 84(6):2022–9. 10.1016/j.wneu.2015.08.039 26342779

[12] Zhao CM Chen Q Zhang Y Huang AB Ding WY Zhang W. Anterior cervical discectomy and fusion versus hybrid surgery in multilevel cervical spondylotic myelopathy: a meta-analysis. Medicine (Baltimore). (2018) 97(34):e11973. 10.1097/MD.0000000000011973 30142827 PMC6113029

[13] de Oliveira Vilaça C Orsini M Leite MA de Freitas MR Davidovich E Fiorelli R Cervical spondylotic myelopathy: what the neurologist should know. Neurol Int. (2016) 8(4):6330. 10.4081/ni.2016.6330 27994827 PMC5136752

[14] Ding X Pan Z Ma Z Ge Z. Clinical application of evoked potentials in the operation of cervical spondylotic myelopathy with different imaging. Contrast Media Mol Imaging. (2022) 2022:4154278. 10.1155/2022/4154278 36299827 PMC9576426

[15] Peng D Ma Y Lei B. Clinical and radiological outcomes of anterior approach microscopic surgery for the pincer mechanism in cervical spondylotic myelopathy. Biomed Res Int. (2019) 2019:9175234. 10.1155/2019/9175234 31016204 PMC6446116

[16] Xia C Shi F Chen C Lv J Chen Q. Clinical efficacy and safety of anterior cervical decompression versus segmental fusion and posterior expansive canal plasty in the treatment of multilevel cervical spondylotic myelopathy. J Healthc Eng. (2022) 2022:7696209. 10.1155/2023/9865031 35449847 PMC9017450

[17] Sun B Han Q Sui F Zhang A Liu Y Xia P Biomechanical analysis of customized cage conforming to the endplate morphology in anterior cervical discectomy fusion: a finite element analysis. Heliyon. (2023) 9(1):e12923. 10.1016/j.heliyon.2023.e12923 36747923 PMC9898605

[18] Zhang J Cui C Liu Z Tong T Niu R Shen Y. Predisposing factors for poor outcome of surgery for cervical spondylotic amyotrophy: a multivariate analysis. Sci Rep. (2016) 6:39512. 10.1038/srep39512 27991596 PMC5171638

[19] Di Martino A Papalia R Albo E Cortesi L Denaro L Denaro V. Cervical spine alignment in disc arthroplasty: should we change our perspective? Eur Spine J. (2015) 24(Suppl 7):810–25. 10.1007/s00586-015-4258-6 26441258

[20] Ament JD Yang Z Nunley P Stone MB Lee D Kim KD. Cost utility analysis of the cervical artificial disc vs fusion for the treatment of 2-level symptomatic degenerative disc disease: 5-year follow-up. Neurosurgery. (2016) 79(1):135–45. 10.1227/NEU.0000000000001208 26855020 PMC4900425

[21] Qureshi SA McAnany S Goz V Koehler SM Hecht AC. Cost-effectiveness analysis: comparing single-level cervical disc replacement and single-level anterior cervical discectomy and fusion: clinical article. J Neurosurg Spine. (2013) 19(5):546–54. 10.3171/2013.8.SPINE12623 24010896

[22] Lu VM Zhang L Scherman DB Rao PJ Mobbs RJ Phan K. Treating multi-level cervical disc disease with hybrid surgery compared to anterior cervical discectomy and fusion: a systematic review and meta-analysis. Eur Spine J. (2017) 26(2):546–57. 10.1007/s00586-016-4791-y 27679431

[23] Xiong Y Xu L Yu X Yang Y Zhao D Hu Z Comparison of 6-year follow-up result of hybrid surgery and anterior cervical discectomy and fusion for the treatment of contiguous two-segment cervical degenerative disc diseases. Spine (Phila Pa 1976). (2018) 43(20):1418–25. 10.1097/BRS.0000000000002639 29547460

[24] Ji GY Oh CH Shin DA Ha Y Yi S Kim KN Artificial disk replacement combined with fusion versus 2-level fusion in cervical 2-level disk disease with a 5-year follow-up. Clin Spine Surg. (2017) 30(5):E620–e7. 10.1097/BSD.0000000000000316 28525488

[25] Kang L Lin D Ding Z Liang B Lian K. Artificial disk replacement combined with midlevel ACDF versus multilevel fusion for cervical disk disease involving 3 levels. Orthopedics. (2013) 36(1):e88–94. 10.3928/01477447-20121217-24 23276359

[26] Maldonado CV Paz RD Martin CB. Adjacent-level degeneration after cervical disc arthroplasty versus fusion. Eur Spine J. (2011) 20(Suppl 3):403–7. 10.1007/s00586-011-1916-1 21796395 PMC3175825

[27] Xu Z Rao H Zhang L Li G Xu Z Xu W. Anterior cervical discectomy and fusion versus hybrid decompression and fusion for the treatment of 3-level cervical spondylotic myelopathy: a comparative analysis of cervical sagittal balance and outcomes. World Neurosurg. (2019) 132:e752–e8. 10.1016/j.wneu.2019.08.022 31415890

[28] Fan X-W Wang Z-W Gao X-D Ding W-Y Yang D-L. The change of cervical sagittal parameters plays an important role in clinical outcomes of cervical spondylotic myelopathy after multi-level anterior cervical discectomy and fusion. J Orthop Surg Res. (2019) 14(1):1–8. 10.1186/s13018-018-1031-7 31829200 PMC6907178

[29] Liu T Tian S Zhang J He M Deng L Ding W Comparison of cervical sagittal parameters among patients with neck pain and patients with cervical spondylotic radiculopathy and cervical spondylotic myelopathy. Orthop Surg. (2023). 10.1111/os.13951 PMC10834188 38093558

[30] Harrison DE Harrison DD Janik TJ Jones EW Cailliet R Normand M. Comparison of axial and flexural stresses in lordosis and three buckled configurations of the cervical spine. Clin Biomech. (2001) 16(4):276–84. 10.1016/S0268-0033(01)00006-7 11358614

[31] Teo AQA Thomas AC Hey HWD. Sagittal alignment of the cervical spine: do we know enough for successful surgery? J Spine Surg. (2020) 6(1):124. 10.21037/jss.2019.11.18 32309651 PMC7154352

[32] Kim SW Shin JH Arbatin JJ Park MS Chung YK McAfee PC. Effects of a cervical disc prosthesis on maintaining sagittal alignment of the functional spinal unit and overall sagittal balance of the cervical spine. Eur Spine J. (2008) 17(1):20–9. 10.1007/s00586-007-0459-y 17721713 PMC2365535

[33] Huang Y Lan Z Xu W. Analysis of sagittal alignment parameters following anterior cervical hybrid decompression and fusion of multilevel cervical spondylotic myelopathy. BMC Musculoskelet Disord. (2019) 20(1):1. 10.1186/s12891-018-2378-y 30611236 PMC6320600

[34] Le Huec J Thompson W Mohsinaly Y Barrey C Faundez A. Sagittal balance of the spine. Eur Spine J. (2019) 28:1889–905. 10.1007/s00586-019-06083-1 31332569

[35] Kim C-W Hyun S-J Kim K-J. Surgical impact on global sagittal alignment and health-related quality of life following cervical kyphosis correction surgery: systematic review. Neurospine. (2020) 17(3):497. 10.14245/ns.2040476.238 33022154 PMC7538364

[36] Protopsaltis TS Scheer JK Terran JS Smith JS Hamilton DK Kim HJ How the neck affects the back: changes in regional cervical sagittal alignment correlate to HRQOL improvement in adult thoracolumbar deformity patients at 2-year follow-up. J Neurosurg Spine. (2015) 23(2):153–8. 10.3171/2014.11.SPINE1441 25978077

[37] Rao H Huang Y Lan Z Xu Z Li G Xu W. Does preoperative T1 slope and cervical lordosis mismatching affect surgical outcomes after laminoplasty in patients with cervical spondylotic myelopathy? World Neurosurg. (2019) 130:e687–e93. 10.1016/j.wneu.2019.06.193 31279919

[38] Sakamoto R Nakamoto H Yoshida Y Ohtomo N Nagata K Kato S Does T1 slope minus cervical lordosis mismatch affect surgical outcomes of cervical laminoplasty in the absence of preoperative severe kyphosis? BMC Musculoskelet Disord. (2022) 23(1):1–6. 10.1186/s12891-022-05755-2 36008857 PMC9404666

[39] Chen S Deng Y Liu H Wu T Huang K He J Cervical sagittal balance after consecutive three-level hybrid surgery versus anterior cervical discectomy and fusion: radiological results from a single-center experience. J Orthop Surg Res. (2023) 18(1):1–10. 10.1186/s13018-022-03481-y 37165448 PMC10170693

